# The interplay among mental and physical health in patients with psoriasis: results from an observational study

**DOI:** 10.1192/j.eurpsy.2025.1224

**Published:** 2025-08-26

**Authors:** S. Cipolla, P. Catapano, A. Cirino, D. D’Amico, A. Volpicelli, V. De Santis, M. Luciano, F. Perris, F. Catapano

**Affiliations:** 1 Department of Psychiatry, University of Campania “L. Vanvitelli”, Naples, Italy

## Abstract

**Introduction:**

Psoriasis is a chronic skin condition affecting 2-3% of the global population and is often associated with various comorbidities, including psychiatric disorders. The psychological burden of psoriasis is substantial and multifaceted. Patients frequently experience stigma, social isolation, and reduced self-esteem due to the visible and often disfiguring nature of the disease. These psychosocial stressors can precipitate or exacerbate psychiatric conditions such as anxiety and depression.

**Objectives:**

This study aims to identify the factors influencing psychopathological symptoms in patients with psoriasis, with a specific focus on the mediation role of physical and mental health on psychopathological distress in patient with psoriasis.

**Methods:**

This observational study included 112 patients diagnosed with psoriasis. Dermatological and psychiatric evaluations were performed using Psodisk, the Hamilton Anxiety Rating Scale (HAM-A), the Hamilton Depression Rating Scale (HAM-D), the Symptom Checklist-90-Revised (SCL-90-R), and the 36-Item Short Form Health Survey (SF-36). Descriptive statistics, correlation analysis, mediation models were used for data analysis. To develop the mediation models, Psodisk subscales were used as independent variables, and the Global Severity Index (GSI) of SCL-90-R as the outcome variable; both physical (PCS) and mental (MCS) components of the SF-36 were used as mediators.

**Results:**

The sample consisted predominantly of middle-aged males (mean age 48.91 years). Females (p < 0.001), patients with arthritis (p < 0.05), and those leading a sedentary lifestyle (p < 0.05) exhibited higher scores for anxiety and depression. Psodisk subscales showed a significant correlation with psychiatric symptoms and QoL measures (p < 0.001). The mediation analysis revealed that the mental component of quality of life (MCS) had a statistically significant mediating effect in all models, being the only mediator for the effect of health, pain, and sleep domains of the Psodisk on GSI (p < 0.001; R^2^ = 0,2). Furthermore, when total Psodisk score was used as independent variable, MCS mediating effect on GSI was also significant (p < 0,001; R^2^ = 0,32) with a non-significant role of PCS.

**Conclusions:**

This study highlights the complex relationship between psoriasis, psychiatric comorbidities, and QoL. Mediation analysis shows that mental component of quality of life can play a more significant mediating role than physical alterations in determining psychiatric symptoms in patients with psoriasis. All psoriasis patients, regardless of physical changes, should be considered at risk for developing psychiatric symptoms, and direct collaboration between general practitioners, dermatologists, and psychiatrists may be able to identify the most vulnerable patients and limit the onset of severe mental disorders.

**Disclosure of Interest:**

None Declared

